# Sensory Profile and Physico-Chemical Properties of Artisanal Honey from Zulia, Venezuela

**DOI:** 10.3390/foods9030339

**Published:** 2020-03-14

**Authors:** Deyanira Araujo, Pilar Ruiz Pérez-Cacho, Salud Serrano, Rafaela Dios-Palomares, Hortensia Galán-Soldevilla

**Affiliations:** 1 Departamento de Bromatología y Tecnología de los Alimentos, Campus de Rabanales, Universidad de Córdoba, 14071 Córdoba, Spain; bt2archd@uco.es (D.A.); pilar.ruiz@uco.es (P.R.P.-C.); bt2sejis@uco.es (S.S.); 2 Departamento de Estadística, Econometría, Investigación Operativa, Organización de Empresas y Economía, Campus de Rabanales, Universidad de Córdoba, 14071 Córdoba, Spain; ma1dipar@uco.es

**Keywords:** multifloral honey, tropical honey, sensory analysis, physico-chemical parameters

## Abstract

The physico-chemical parameters and the sensory profile were determined in honeys from apiaries of the Mara and Maracaibo of Zulia State (Venezuela). The analysis of variance showed that there were no significant differences in the mean value between apiaries for most of the physicochemical parameters and sensory attributes. The obtained value for pH (3.58–4.08), free acidity (30.9–36.0 meq/kg), lactone acidity (9.0–14.3 meq/kg), total acidity (42.1–46.0 meq/kg), moisture content (19.1–20.0%), diastase activity (8.11–12.7 ºG), colour intensity (41.5–86.6 mm Pfund), hydroxymethylfurfural (15.7–26.0 mg/kg), and electrical conductivity (0.33–0.52 mS/cm) were within the criteria set by international quality regulations. The sensory profile of these honeys is characterized by being amber in colour, with a floral, acid fruit, balsamic and animal odour/aroma, a sweet, slightly acid taste, and by being fluid and of a medium persistence.

## 1. Introduction

Honey is a natural product that needs no transformation for its consumption. Thus, assessing its quality consists of measuring its authenticity, including descriptive (botanical and geographical origin) and production factors, up to terms, like natural, organic, raw, or non heat-treated [1,2].

Sensory characteristics of honeys basically depend on its geographical origin, with each honey type presenting its own sensory and differentiating characteristics, which define its intrinsic quality [1,2,3,4]. Therefore, sensory analysis of honey might be used as a complement to physico-chemical and pollen analyses [5,6]. There is a growing awareness on the part of the food industry that its sensory characteristics are decisive in establishing the acceptance of a food by the consumer. Consequently, their control is of great importance from both a technological and an economic perspective if it is desired to launch a competitive product on the market.

Venezuela is not known abroad for the quality of its honey, although, being a tropical country and the fact of its possessing over 400,000 Km^2^ suitable for apiculture, augurs a great potential for growth with regard to the marketing of this product. Zulia is a geographical region that produces a honey that is highly appreciated by local consumers, with its quality being based on this region’s rich apicultural flora, with an abundant proliferation of pollen-producing and melliferous species of the *Leguminosae*, *Rutaceae*, *Anacardiaceae*, and *Myrtaceae* families, which include *Acasias*, *Citrus*, *Mango*, *Merey*, and *Eucaliptus* [7,8,9,10]. The Apiarist Association of the State of Zulia (AZUAPI) needed to establish a quality seal that permitted it to protect its honey products and preserve the economic and cultural activity of the population, thus guaranteeing the authenticity of those products and providing legal protection from the production of honeys from other geographical areas.

The purpose of this work was to establish values for authentic Zulia honey and determine whether it meets national and international compositional standards of honey specifications and the sensory profile of these honeys, since there are very little data available on the composition and sensory attributes of Venezuelan honey [9,11].

## 2. Materials and Methods

### 2.1. Samples

**Figure 1:**
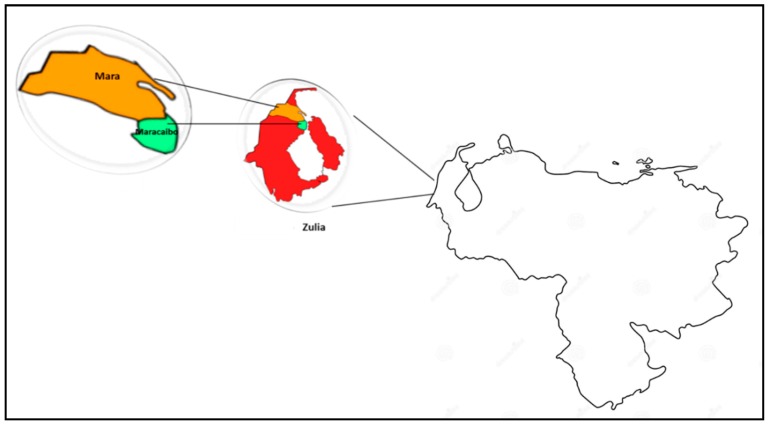
Geographical locations of Mara and Maracaibo municipalities in Zulia State (Venezuela).

Thirty-two *Apis mellifera* honey samples of multifloral types from Mara and Maracaibo municipalities (Figure 1) at Zulia state (western region of Venezuela) were directly obtained from five apiarists during four successive years. The samples were provided in sterile glass jars (1 kg), processed as soon as they were obtained, and stored at 4 °C until fully analysed.

### 2.2. Physico-Chemical Analysis

The *pH* was potentiometrically measured according to Bogdanov et al. [12], while using a Crison pH meter (Crison Instruments, Barcelona, Spain). *Free*, *lactonic, and total acidity* (FA, LA, TA) were analysed in accordance with AOAC [13] method no. 962.19. The *glucose oxidase* (GOx) was determined in accordance with Kerkvliet [14], using Merckoquant test (Merck, Darmstadt, Germany). *Water content* (H) was determined following Chataway [15] and Wedmore [16], a method established by the Codex Alimentarius Commission [17]. For *diastase activity* (DA) determination, the procedure of Siegenthaler [18], as modified by Bogdanov [12], was used. *Hydroxymethylfurfural* (HMF) was ascertained in accordance with White [19]. A Pharmacia Biotech Ultrospec-3000 spectrophotometer (Pharmacia Biotech Ltd., Cambridge, UK) was used for both tests. The *colour intensity* of honey samples was measured following the Pfund classifier. Briefly, homogeneous honey samples that were devoid of air bubbles were transferred into a cuvette with a 10-mm light path and then placed in a Lovibond 2000 apparatus comparing them with a standard colour honey wheel. *Electrical conductivity* (EC) was measured using a Crison model 524 conductometer (Crison Instruments, Barcelona, Spain), according to Vorwohl [20]. All of the analyses were carried out in duplicate.

### 2.3. Sensory Analysis

#### 2.3.1. Sample Preparation

Following Piana et al. [21], 40 g of each sample was put into a glass vial and covered with a watch glass for sensory analysis. The samples were prepared one hour before tasting to achieve the equilibrium of the headspace and they were served at 20 °C. Three to four samples, labelled with three-digited random numbers, were served, one at a time, over a session. Mineral water was used to cleanse the palate between samples.

#### 2.3.2. Assessors

Ten (three men, seven women) highly trained panellists from the Sensory Laboratory at the University of Córdoba (Spain), aged from 27 to 60, participated in this study. They were selected and trained following international standards (ISO) [22,23,24]. Their selection was based on detection, recognition, and discrimination tests, and on the ability of candidates to memorize and communicate sensory impressions. These panellists had prior experience in the sensory evaluation of different products and they had undergone specific training in honeys [1,2,3,25]. Testing was carried out in the sensory laboratory that was equipped with a round table for training sessions and individual booths, in accordance with the ISO [26]. All of the analyses were conducted in the morning (10 a.m.–12.p.m.).

#### 2.3.3. Sensory Profile

The followed methodology is based on ISO [24] and on an adaptation of that the one proposed by Piana et al. [21] established by the International Honey Commission. The profile sheet was made following Galan-Soldevilla et al. [3]. Twenty-four sensory attributes were evaluated, one for appearance, 22 for flavour (14 for odour/aroma, four basic tastes, three trigeminal sensations and persistence), and one for texture. The attributes were evaluated on a non-structured scale of 10 cm. In each sample, first its odour was analysed, and then its colour intensity and its fluidity, next its aroma, basic tastes and trigeminal sensations, and last its persistence.

### 2.4. Statistical Analysis

All of the statistical treatments were done with the SPSS 17.A program. A basic descriptive statistical analysis was performed to obtain an overall impression of the data and discover possible trends that might be of interest in subsequent analyses (mean and standard deviation). One-way ANOVA was applied for each physicochemical parameter and sensory attribute to test mean differences between apiaries, followed by Tukey test at 95% confidence level (*p* < 0.05). In addition, the one-way ANOVA was applied for each sensory attribute to test mean differences between assessors and a Levene statistic was performed to test for heteroscedasticity among the assessors.

We made a hypothesis contrast to make a comparative analysis of the physicochemical parameters and sensory attributes of the Venezuelan honeys with honeys from other parts of the world, as follows:

Let Xv be the variable that measures one characteristic of the Venezuelan honey, with a mean µv that is unknown and estimated through the sample mean $\bar{X_{V}}$, an unknown variance being estimated by the quasi-variance $\bar{S_{v}^{2}}$, on the basis of a sample size n. Let Xo be the same characteristic of honey from another country, with a mean = µo, which we know from the bibliography. Let us contrast the following null hypothesis:

H_0_: µv = µo: The Venezuelan honey’s characteristic is the same as that of the other country
H_1_: µv ≠ µo: The Venezuelan honey’s characteristic is different to that of the other country.

To make the contrast a confidence interval of γ (e.g., 0.95) was estimated for µv, so that:

$$P\left(Ia\leq \mu_{V}\leq I_{b}\right)=\gamma$$

$$P\left(\bar{X_{v}}-t_{\frac{\alpha }{2}}\frac{\bar{S_{v}}}{\sqrt{n}}\leq \mu_{V}\leq \bar{X_{v}}+t_{\frac{\alpha }{2}}\frac{\bar{S_{v}}}{\sqrt{n}}\right)=\gamma$$

α = 1 − γ (e.g., 0.05) and $t_{\alpha /2}$ being the value of the Student-t variable with *n* − 1 degrees of freedom, corresponding to the percentile (1 − α/2).

If the value of µo is comprised between Ia and Ib, the null hypothesis Ho is accepted. It can be concluded that the characteristic of the Venezuelan honey is the same as that of the other country’s honey, with a probability equal to γ. If not, it is assumed that the honeys are different with respect to that characteristic.

## 3. Results and Discussion

### 3.1. Physico-Chemical Characteristics of Honey Samples

Table 1 presents the means, the standard deviations, and the analysis of variance between apiaries for the physiochemical parameters studied, and Table 2 gives a comparison of relevant published data of multifloral honey parameters from different regions of South America. The results show that there were no significant differences between apiaries (*p* < 0.05, Table 1), except for their pH (*p* < 0.05) and instrumental colour (*p* < 0.001).

With respect to the pH, the latter varied at between 3.6 and 4.1 (Table 1), with a mean value of 3.8 and a confidence interval (95%) of between 3.7 and 3.9 (Table 2), a value that corresponds to honeys of a floral origin. If we compare this result with those that were published by other authors for multifloral honeys (Table 2), it is observed that only the Venezuelan honeys from Zulia [9] and the Mexican ones [27] displayed the mean values included within the confidence interval of the honeys in our study; honeys from Argentina [28,29], Uruguay [30], and Brazil [31] had slightly lower values. In addition, the mean pH values of honey samples were lower than those that were previously reported in other tropical countries, such as India [32,33] or Thailand [34].

Colour in honeys varies from clear to dark amber and it is characteristic of its floral source [35]. In our study, the colour intensity varied at between 41.6 and 86.6 (Table 1), with a mean value of 56.5 and a confidence interval (95%) of between 49.3 and 63.8 (Table 2), which correspond to honeys medium amber in colour, and those values were similar to those that were found for Argentina ones [30,31] and Ethiopian honeys [35].

*Acidity (free, lactone, and total)* is an important quality criterion. Honey fermentation causes an increase in acidity and, accordingly, a maximum free acidity value has been fixed with a maximum of 50 milliequivalents/kg in the CAC [17] and UE standard [36], and 40 milliequivalents/kg in the COVENIN Standard [37]. The results in Table 2 show that free acidity is of a mean value of 33.4 meq/Kg and it has a confidence interval (95%) of between 31.7 and 35.2, which suggests that there was no undesirable fermentation in the honey samples, although those values are higher than those that were reported by Isla et al. [28], Silvano et al. [38], and Baroni et al. [29], in Argentina honeys, or by Kumar et al. [33], in Indian ones. The lactone acidity presented a confidence interval (95%) of between 9.20 and 11.0, with a mean of 10.1 meq/Kg (Table 2), a slightly higher value than that reported by Baroni et al. [29], for honey from Córdoba, Argentina. With respect to total acidity, this gave a confidence interval (95%) of between 41.6 and 45.5, with a mean value of 43.5 meq/Kg (Table 2). Other authors’ results were found to be outside this total acidity range [9,29,33]. The enzyme *glucose oxidase* catalyzes the oxidation of glucose to H_2_O_2_ and gluconic acid (or its lactone). White et al. [39] demonstrated that the antibacterial effects of inhibine result from the accumulation of hydrogen peroxide (H_2_O_2_), which is an enzyme that is produced by a natural glucose oxidase system in honey, and as a by-product of glucose oxidase activity in honey or sugar dilutions. Hydrogen peroxide formation is partially responsible, alongside other components, for the antibacterial effect of honey. The mean value of the activity that was obtained in our study was 4.84 µg H_2_O_2_/g honey/h, with a confidence interval (95%) of 3.97 to 5.72 (Table 2), with no values being published in the bibliography being found for tropical multifloral honeys.

*Moisture content* is the only honey composition criterion that forms part of the Honey Standard [17]. It depends on the climate conditions, on the handling of the beehive, and on the honey’s storage conditions, and it has influence on honey conservation, since high moisture values favour its fermentation. The moisture results showed a confidence interval (95%) of between 19.4 to 19.9%, with a mean value of 19.6% (Table 2), and they were in concordance with the maximum limit that was set by COVENIN (≤20%) and CAC (<21%). These observations are in agreement with the findings of various researchers for Venezuelan multifloral honeys [9], but their values were higher than those of Argentinian [28,29,38,40], Uruguayan [30], and Brazilian multifloral honeys [31], and lower than Mexican ones [27].

Honey *diastase activity* is a quality factor, being influenced by honey storage and heating, and is thus an indicator of honey freshness and overheating [41]. The diastase activity that is found in our study presents a confidence interval (95%) of from 8.71 to 10.5, with a mean value of 9.59 (Table 2), higher values than the 8 Göthe units that are required as quality criteria by the CAC [17]. The consulted works report higher values than those that were obtained for this parameter in the current study [29,31,40].

The high *HMF* content of honey indicates poor storage conditions and overheating during processing as fresh honey does not contain HMF [41]. The EU standard [36] and the Codex [17] demand a maximum of 40 mg/kg. However, a maximum of up to 80 mg/kg [36] is permitted for honeys from tropical climate regions and their blends. In the current research, the HMF content displayed a confidence interval (95%) of from 22.4 to 29.6 mg/kg with a mean value of 26.0 mg/kg (Table 2), which was below the maximum acceptable level that was established by CAC and EU, and similar to that reported by Baroni et al. [29] for southern Córdoba honeys in Argentina. These values are lower than those that were reported by other authors [31,40] and higher than those in other studies [28,30,33,38].

The *electrical conductivity* of honey is closely related to the concentration of mineral salts and organic acids in it. The EC results, with a confidence interval (95%) of 0.37 to 0.47 mS/cm, and a mean value of 0.42 (Table 2), were below the maximum allowed by the EU standard [36], and the Codex [17] of 0.8 mS/cm. These values are similar to the findings in honeys from Arana Delta and Islands in Argentina [40], but higher than those that were reported in multifloral honeys from Lara and Yaracuy in Venezuela [11], Buenos Aires, Northwest and Espinal in Argentina [28,38,40], and Tabasco in Mexico [27], and lower than those of Patagonian forest honeys in Argentina [40] and Uruguayan honey [30].

### 3.2. Sensory Profile

One-way ANOVA was performed for each sensory attribute with the assessor as the factor. The results of the analyses showed that the panel worked as a whole (*p* value of between 0.65 for sweet taste and 0.98 for persistence). Additionally, homoscedasticity was accepted for each sensory variable on the base of the Levene statistic (*p* value of between 0.76 for acid taste and 1 for colour intensity).

Table 3 presents the means, standard deviations, and the analysis of variance between apiaries for the sensory attributes studied. The results show that there was a single qualitative profile for the five apiaries analysed, with all of the honeys being amber in colour, with floral, acid fruit, balsamic and animal olfactory notes, a sweet but a slightly acid taste, and are fluid, and of a medium persistence. Additionally, some apiaries had olfactory notes of caramel (apiaries 1 and 2), of smoke (apiaries 3 and 4), and had a salty taste (apiaries 2 and 3). For common sensory attributes, the results in all of the honeys show that there were only significant differences between apiaries for colour intensity (*p* < 0.001), floral odour (*p* < 0.01), acid fruit odour (*p* < 0.001), and balsamic aroma (*p* < 0.01).

If we compare our findings with other research works, we found that there are very few on multifloral tropical honeys [11,28,32,33] and most of these works only give information on the appearance (colour), texture (granularity and fluid) and basic tastes (sweet and acid) and not on the odour/aroma attribute characterizing them. Only Anupama et al. [32] described Indian honeys in odour/aroma terms (flowery, fruity, waxy, jaggery-like, caramelised, chemical and fermented).

Table 4 gives a comparison of relevant published data of multifloral honey sensory attributes from different regions of South America.

*Colour intensity* values, with a confidence interval (95%) of 4.9 to 5.8 and a mean value of 5.4 (Table 4), are similar to the findings of honeys from Lara and Yaracuy in Venezuela [11] and from India [32], but higher than those that were reported in multifloral honeys from Northern India [33]. *Overall odour intensity* reported in this work, with a confidence interval (95%) of 5.9 to 6.4 and a mean value of 6.1 (Table 4), was lower than that found in honeys from Lara and Yaracuy in Venezuela [11]. *Floral odour* found in our study, with a confidence interval (95%) of 4.2 to 4.9 and a mean value of 4.5 (Table 4), was similar to those that were reported by Silvano et al. [38] for honeys from Agricultural zone in Argentina and for some Indian commercial honeys [32], and higher than those reported by Kumar et al. [33] for multifloral honeys. *Fruit odour*, with a confidence interval (95%) of 2.3 to 3.4 and a mean value of 2.8 (Table 4), agreed with some commercial honeys from India [32]. The *sweet taste* presented a confidence interval (95%) of from 5.5 to 6.3, with a mean value of 5.9 (Table 4), a value that is similar to those reported by Anupama et al. [32] for some commercial Indian honeys, higher than those reported by Silvano et al. [38] for the Buenos Aires ones and lower than Northern Indian ones [33]. *Acid taste*, with a confidence interval (95%) of 3.5 to 4.42 and a mean value of 3.9 (Table 4), was higher than those that were reported by Anupama et al. [32] for commercial Indian honeys. Finally, *fluidity* with a mean value of 6.1 and confidence interval (95%) of 5.5 to 6.6 (Table 4), was similar to those that were reported by Anupama et al. [32] for some commercial Indian honeys and higher than those from Venezuela [11], Argentina [38], and Northern India [33].

## 4. Conclusions

The results suggest that Zulia honeys are of good quality, as they comply with the compositional standards established by national and international regulations and had a complex sensory profile in odour/aroma terms (floral, fruit, balsamic, and animal) highly appreciated by local consumers. These results could help the beekeepers of the state of Zulia (Venezuela) to protect their honey from those of other geographical areas.

## Figures and Tables

**Table 1:** Descriptive measures (means values and standard deviation) and analysis of variance (apiarist) of physicochemical parameters (probability values).

	pH	FA (meq/Kg)	LA Meq/Kg)	TA (meq/kg)	GOx (µg H_2_O_2_/g honey/h)	H (%)	DA (ºG)	Colour (mm Pfund)	HMF (mg/kg)	EC (mS/cm)
**Apiarist 1**										
	3.62 ± 0.20 ^a^	31.7 ± 8.98	14.3 ± 11.1	42.1 ± 10.9	5.50 ± 3.76	20.0 ± 0.81	9.24 ± 2.39	41.5 ± 25.7 ^a^	26.0 ± 7.65	0.33 ± 0.05
**Apiarist 2**										
	3.87 ± 0.15 ^ab^	33.8 ± 7.79	9.90 ± 2.28	43.6 ± 7.70	5.31 ± 3.75	19.6 ± 0.59	10.0 ± 4.00	58.8 ± 20.3 ^ab^	27.9 ± 12.5	0.43 ± 0.21
**Apiarist 3**										
	4.08 ± 0.42 ^b^	36.0 ± 5.06	9.95 ± 4.08	46.0 ± 5.80	4.00 ± 3.16	19.3 ± 0.94	8.55 ± 2.83	86.6 ± 29.1 ^b^	19.1 ± 11.1	0.49 ± 0.25
**Apiarist 4**										
	3.58 ± 0.15 ^a^	30.9 ± 2.04	11.3 ± 1.55	42.2 ± 1.17	4.38 ± 3.47	19.1 ± 1.01	12.7 ± 5.10	44.4 ± 7.65 ^a^	15.7 ± 3.78	0.52 ± 0.12
**Apiarist 5**										
	3.98 ± 0.33 ^b^	35.8 ± 5.04	9.00 ± 2.74	44.8 ± 4.15	4.00 ± 3.16	19.4 ± 1.21	8.11 ± 2.51	62.8 ± 35.7 ^ab^	16.3 ± 9.93	0.42 ± 0.27
***p***	0.001	ns	ns	ns	ns	ns	ns	0.001	ns	ns

Values followed by the same letter within the same column are not significantly different (*p* > 0.05) according to Tukey’s Multiple Range Test. FA = Free acidity; LA = Lactonic acidity; TA = Total acidity; GOx = Glucose Oxidase; H = Moisture; Diastase activity = DA; HMF = Hydroxymethylfurfural; EC = Electrical conductivity. ns = not significant

**Table 2:** Comparison of physico-chemical parameter of multifloral honeys from South America.

		**pH**	**FA (meq/Kg)**	**LA** **(meq/Kg)**	**TA** **(meq/kg)**	**GOx** **(µg/g/h)**	**H** **(%)**	**DA** **(ºG)**	**Colour** **(mm Pfund)**	**HMF (mg/kg)**	**EC** **(mS/cm)**
**Current study data**											
Mean		3.8	33.4	10.1	43.5	4.84	19.6	9.59	56.5	26.0	0.42
(I_a_ 0.95–I_b_ 0.95)		(3.7–3.9)	(31.7–35.2)	(9.20–11.0)	(41.6–45.5)	(3.97–5.72)	(19.4–19.9)	(8.71–10.5)	(49.3–63.8)	(22.4–29.6)	(0.37–0.47)
**Venezuela**											
[9]	Zulia	3.7	NA	NA	37.3	NA	19.3	NA	NA	NA	NA
[11]	Lara	3.4	NA	NA	NA	NA	20.2	NA	NA	NA	0.21
	Yaracuy	3.3	NA	NA	NA	NA	17.6	NA	NA	NA	0.05
**Argentina**											
[28]	NW	3.5	24.8	NA	NA	NA	16.5	NA	83	12.5	0.33
[38]	BA-Meadows	NA	<18.0	NA	NA	NA	17.0	NA	<50.0	<7.0	<0.24
	BA-Hills	NA	<18.0	NA	NA	NA	17.0	NA	<60.0	<3.0	
	BA-Agricultural	NA	<18.0	NA	NA	NA	17.0	NA	<70.0	<7.0	
[40]	Patagonian forest	NA	NA	NA	NA	NA	15.7	26.4	62.0	7.0	0.53
	Espinal	NA	NA	NA	NA	NA	17.9	26.0	26.0	8.2	0.27
	Parana Delta and Islands	NA	NA	NA	NA	NA	17.6	22.8	81.3	33.9	0.40
[29]	Córdoba N	4.1	22.8	2.6	25.4	NA	17.4	17.9	NA	5.4	NA
	Córdoba S	3.6	20.4	3.8	24.4	NA	18.3	20.3	NA	29.4	NA
**Uruguay**											
[30]		3.2	NA	NA	NA	NA	18.0	NA	99	5.25	0.60
**México**											
[27]	Tabasco	3.7	NA	NA	NA	NA	20.9	NA	NA	NA	0.25
**Brasil**											
[31]		3.6	NA	NA	32.9	NA	19.1	12.6	NA	35.9	NA

FA = Free acidity; LA = Lactonic acidity; TA = Total acidity; GOx = Glucose Oxidase; H = Moisture; Diastase activity = DA; HMF = Hydroxymethylfurfural; EC = Electrical conductivity; NA: not available.

**Table 3:** Descriptive measures (means values and standard deviation) and analysis of variance (apiarist) of sensory attributes (probability values).

	Apiarist 1	Apiarist 2	Apiarist 3	Apiarist 4	Apiarist 5	*p*
Colour Int.	4.8 ± 1.6 ^a^	6.3 ± 1.2 ^b^	6.3 ± 1.7 ^b^	3.1 ± 0.5 ^a^	5.8 ± 1.3 ^b^	0.001
Odour overall int.	5.8 ± 1.1	6.4 ± 1.2	6.2 ± 0.4	6.2 ± 0.3	6.1 ± 1.0	ns
o_Floral	3.8 ± 1.6 ^a^	4.4 ± 0.8 ^ab^	5.2 ± 0.7 ^b^	4.9 ± 0.6 ^ab^	5.3 ± 0.9 ^b^	0.01
o_Acid fruit	3.6 ± 1.0 ^a^	4.7 ± 0.5 ^b^	3.1 ± 1.2 ^a^	4.4 ± 0.6 ^b^	3.2 ± 0.8 ^a^	0.001
o_Balsamic	-	3.5 ± 0.6	-	-	5.5 ± 0.9	-
o_Caramel	4.2 ± 1.1	-	-	-	-	-
o_Animal	5.3 ± 1.2	4.2 ± 1.3	5.1 ± 1.2	5.2 ± 0.6	-	-
o_Smoke	-	-	5.7 ± 0.7	4.5 ± 0.3	-	-
Aroma overall int.	6.2 ± 1.0	6.6 ± 0.9	6.2 ± 1.3	5.7 ± 0.5	6.6 ± 0.8	ns
a_Acid fruit	4.9 ± 1.0	4.7 ± 0.9	5.0 ± 0.6	4.6 ± 0.5	-	-
a_Balsamic	4.9 ± 1.2 ^ab^	4.1 ± 1.3 ^a^	4.0 ± 0.6 ^a^	4.5 ± 1.1 ^a^	5.8 ± 1.0 ^b^	0.01
a_Caramel	5.3 ± 0.6	5.8 ± 1.0	-	-	-	-
a_Animal	-	-	-	3.7 ± 0.9	-	-
a_Smoke	-	-	5.7 ± 1.0	4.4 ± 0.5	-	-
Sweet taste	6.2 ± 1.4	6.4 ± 2.0	5.7 ± 0.9	4.4 ± 1.2	6.1 ± 1.8	ns
Acid taste	3.8 ± 1.6	4.5 ± 1.5	3.8 ± 0.8	4.2 ± 1.9	2.7 ± 0.9	ns
Salty taste	-	4.1 ± 1.3	3.2 ± 0.9	-	-	-
Fluidity	6.0 ± 2.5	6.5 ± 2.3	6.1 ± 1.4	4.5 ± 2.23	6.6 ± 1.2	ns
Persistence	5.3 ± 1.3	6.2 ± 1.7	6.0 ± 1.7	6.0 ± 1.1	7.0 ± 1.3	ns

o = odour (orthonasal perception); a = aroma (retronasal perception). Values followed by the same letter within the same column are not significantly different (*p* > 0.05) according to Tukey’s Multiple Range Test.

**Table 4:** Comparison of sensory attributes of multifloral honeys from South America.

		Colour Intensity	Odour Intensity	Floral	Fruit	Sweet Taste	Acid Taste	Fluidity
**Current study data**								
Mean		5.4	6.1	4.5	2.8	5.9	3.9	6.1
(I_a_ 0.95–I_b_ 0.95)		(4.9–5.8)	(5.9–6.4)	(4.2–4.9)	(2.3–3.4)	(5.5–6.3)	(3.5–4.2)	(5.5–6.6)
[11]	Lara	5.1	6.5	NA	NA	NA	NA	5.3
	Yaracuy	5.1	6.5	NA	NA	NA	NA	5.3
[38]	BA-Meadows	NA	NA	2.7	NA	4.8	NA	*6.5
	BA-Hills	NA	NA	3.9	NA	4.2	NA	*6.1
	BA-Agricultural	NA	NA	4.3	NA	4.1	NA	*6.6

NA: not available; * Viscosity values.
